# 

**Published:** 1886-01

**Authors:** 


VOL. VII. ] TWO DOLLARS A YEAR. SINGLE COPIES 60 CENTS. [No. 1.

LIBRARY INDEXED S.G.O.

### THE JOURNAL

OF

### COMPARATIVE MEDICINE AND SURGERY.

EDITED BY

W. A. CONKLIN, PH.D., F. S. BILLINGS, D.V.S.

ASSISTED BY

Prof. ALBERT JOHNE, Royal Veterinary School, Dresden, Germany.
THOMAS B. ROGERS, D.V.S., Veterinary Department, University of Penn'a.
A. W. CLEMENT, V.S., Montreal Veterinary College.
A. T. YOKURA, V.S., Imperial Agricultural School, Japan.

SURGEON GEN'L'S OFFICE LIBRARY

Entered New York Post Office as second class matter.

### JANUARY, 1886—CONTENTS.

WILLIAM R. JENKINS,
VETERINARY PUBLISHER, BOOKSELLER AND PRINTER,
850 Sixth Ave., Cor. 48th Street,
NEW YORK.
LONDON: BAILLIÈRE, TINDALL & COX.

